# Morphometry, hematology, and plasma chemistry of common coot (*Fulica atra*) in Punjab, Pakistan

**DOI:** 10.5455/javar.2025.l898

**Published:** 2025-03-26

**Authors:** Shozab Seemab Khan, Tariq Javed, Muhammad Saleem Khan, Zahid Farooq, Muhammad Wajid

**Affiliations:** 1 Department of Zoology, Faculty of Life Sciences, University of Okara, Okara, Pakistan; 2 Department of Zoology, Cholistan University of Veterinary and Animal Sciences, Bahawalpur, Pakistan

**Keywords:** Migratory birds, hematological, parameters, plasma chemistry, morphometry

## Abstract

**Objective:**

The common coot (*Fulica atra*) is a medium-sized migratory bird wintering at different wetlands in Pakistan. It belongs to the order Gruiformes and family Rallidae. This study aimed to investigate the morphometry, hematology, and blood chemistry of common coots during the winter visit of 2022–2023.

**Materials and Methods:**

Thirty two adult common coots were captured with the help of hunters holding valid licenses from six wetlands in Punjab, Pakistan. Each individual was sexed and subjected to different morphological measurements. Eighteen blood samples (3–5 ml) were collected from the basilic veins of adults for hematology and blood chemistry.

**Results:**

Results revealed that body weight ranged from 378 to 680 gm, with males significantly heavier than females. Length was not significantly different. Males showed dominance in most of the morphometric characteristics compared to females. Regarding hematological parameters and plasma chemistry, there was a sufficient difference between the genders in most of the studied parameters.

**Conclusion:**

The male coot was significantly larger compared to the female, except for total length. Hematology and plasma chemistry showed significant differences between genders.

## Introduction

Total wetland habitats in Pakistan cover approximately 7,800 km², approximately 9.7% of the total area of the country [1]. These wetlands are a habitat in winter for large numbers of birds, including ducks, geese, and swans [2]. About 670 bird species have been reported from Pakistan, and one-third are migratory water birds [3].

The common coot (order Gruiformes, family Rallidae, species *Fulica atra*) is one of the medium-sized migratory birds wintering at different wetlands in Pakistan [4]. The species breeds on its summer grounds in Siberia and other Russian states [5]. It has an all-black body with no white on the under-tail coverts. The beak is pinkish-white in color, topped with a distinct, bright white frontal shield on the forehead [6]. The body plumage is predominantly sooty black on the head and neck, transitioning to a grayish color on the back and flanks. During the flight, a narrow whitish band along the trailing edges of the secondaries becomes visible. The feet of the bird are greenish-gray and equipped with distinctively lobed long toes. The iris of both male and female birds is a vivid crimson. The elongated toes extend beyond the tail tips during flight, while the rounded wings produce rapid, whirring beats; the subtlest smear of the orange-red is visible on the tibia. Notably, there is no seasonal variation in the plumage [7]. No sexual dimorphism, though males are larger than females [8].

The common coot is omnivorous, mainly feeding on submerged weeds, annelids, gastropods, insects, small fish, and eggs [7]. The duration of dives is directly proportional to the depth of the water body, but the frequency of diving per day remains relatively constant. The common coot is a protected species in Pakistan [9]; however, it is recorded as the most common species at Uchhali [10] and Kallar Kahar Lake [11].

Morphometric studies on birds have their importance in understanding geographic variability, developmental stages, and genetic and environmental effects [12]. It plays a key role in conservation. The comparison of morphometric measurements can provide reasonable information on genetic differences between populations throughout their annual cycle [13]. Variations in body size or the size of specific anatomical features influenced mating selection, clutch size, egg size, survival outcomes, and patterns of differential migration [14,15].

Hematological analysis offers valuable insights into various aspects of an individual’s health, including nutritional status, physiological condition, and the presence of pathological disorders [16]. The test also provides information about immunology [17], parasitic infection [18], and exposure to toxic and hazardous substances. Meaningful interpretation of these values can be used as reference values [19]. To identify such influences, it is essential to build baseline data of healthy birds of both sexes [20]. Keeping in view the importance of the water birds, the present study was planned to evaluate the morphometry and hematological parameters of common coots (*F. atra*) collected from six wetlands of Punjab (Pakistan).

## Materials and Methods

### Ethical approval

Ethical approval was taken from the Ethics Committee of the University of Okara, with reference No. UO/DOZ/2023/SSK1.

### Study areas

Samples of common coot were collected from six water bodies distributed in the Punjab (Pakistan).

Tunsa Barrage (30.5129°N, 70.8496°E) is a Ramsar site located on the river Indus, Tehsil Kot Addu, District Muzafargarh, with a lake area of 2,832 hac. The climate is subtropical, with the average lowest temperature in January (4.5°C–6.0°C) and the average maximum in June (41.5°C –43°C).

Chashma Barrage (32.4359°N, 71.3803°E) is located in Tehsil Mianwali with approximately 327,000 hac area. It was declared a wildlife sanctuary in 1974.

Marala Headworks (32.6724°N, 74.4644°E, approximately 1,620 hac lake area located at River Chanab, District Sialkot was declared a Game reserve in 1987.

Head Sulemanki (29.49°NL, 72.33°E) is located on the River Sutlej, with a lake receiving water supply from the Chenab and Ravi rivers.

Balawalnagar district (29.59°N, 73.16°E) holds a complex of many brackish water bodies, providing feeding and resting places for migratory birds.

Faisalabad district (31.25°N, 73.5°E) has the rivers Ravi and Chenab flowing through with wetlands at different places along the run of the rivers.

### Morphometric variables

A total of 32 adult common coots (*F. atra*) were captured with the help of hunters holding valid licenses during field visits between September 2022 and March 2023. Each individual was sexed, anesthetized (diazepam at 0.2 mg/kg), and ketamine HCl at 10 mg/kg and subjected to different morphological measurements, *viz*., body weight (electronic balance; minimum 0.01 gm). The body length was measured from the tail to the beak, while the wingspan was measured from the outer wingtips of both wings to connect them. The primary wing length was measured with wing bend and assessment of the first primary feather extension. The measurement of tail length began at its base point and continued to its tip, where the longest tail feather ended. The tarsal length was measured by examining the distance between the shank and the base of the toes, and the metatarsal length was measured by extending the tape from the ankle joint to the tip of the toenails. Furthermore, the body circumference was measured at the breast section where the area was widest. The measurement of beak length covered the entire visible segment from the tip toward the hairline where the feathers emerge. Head length measurement began at the back of the skull and ended at the bill tip.

### Blood sampling

Eighteen blood samples (3–5 ml) were collected from the basilic vein of adult common coots using 5 ml disposable syringes with 22–25-gauge butterfly needles. Animal ethics were ensured during the process. Collected blood was transferred to a 5 ml blood two vacutainer marked with a unique sample number and placed in a cooler containing ice. One vacutainer, having EDTA (an anticoagulant), was used for hematology, and another vacutainer without EDTA was used for blood serum analysis.

The analysis of the sample was carried out in laboratories. The heparinized blood samples were subjected to hematological analysis, *viz*., TLC, TEC, PVC, Hb, DLC, and PCV using XP-100 Sysmex, Japan. Mean corpuscular volume (MCV), hematological indices, mean corpuscular hemoglobin concentration (MCHC), and mean corpuscular hemoglobin (MCH) were recorded from erythrocyte series values [21]. Serum chemistry tests were carried out using commercial diagnostic kits: total protein through the biuret method, glucose using the enzymatic colorimetric glucose oxidase method, serum urea through the enzymatic colorimetric, endpoint, and Berthelot method, and serum creatinine by the kinetic method-Jaffe reaction [22].

### Statistical analysis

Data were analyzed using standard statistical methods, including mean, SE of the mean, and range. Differences between groups were assessed using an unpaired *t*-test at a 0.05 significance level. Pearson correlation coefficients were calculated to evaluate relationships between variables.

## Results

The body weight was significantly higher (*p < *0.01) in males (633.00 ± 10.670 gm) compared to females (444.56 ± 10.81 gm). Body length was non-significantly different (*p* > 0.05) between male (40.58 ± 0.56 cm) and female (39.39 ± 0.53 cm). The rest of all studied parameters were significantly different and larger or greater in male as compared to female common coots (Table 1).

Regarding hematological parameters, male and female common coots showed sufficient differences in most of the studied parameters except red cell distribution width–SD (RDW-SD) (fl), red cell distribution width–coefficient of variation (RDW-CV) (fl), and MCH (pg) (Table 2).

**Figure 1.**
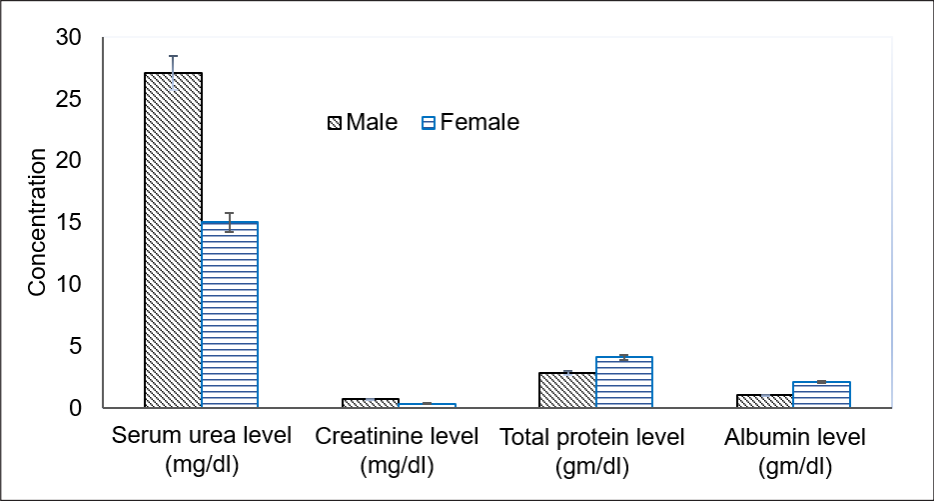
Comparison of biochemical variables in male and female of adult common coot.

**Figure 2.**
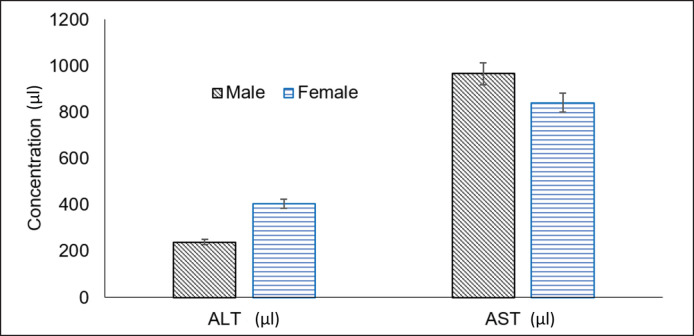
The comparison of biochemical variables in male and female adult common coot.

Serum urea level was significantly (*p < *0.01) higher in males (27.10 ± 0.657 mg/dl) than in females (*n = *8, 15.00 ± 0.906 mg/dl). A similar trend was observed for creatinine level (male: 0.72 ± 0.036 mg/dl, female: 0.36 ± 0.032 mg/dl). The alanine transaminase (ALT) range was significantly (*p < *0.05) lower in males (239.20 ± 47.641 µl) than in females (404.00 ± 41.423 µl). The same was in the case of total protein in males (2.82 ± 0.149 gm/dl) and females (4.11 ± 123.890 gm/dl), and albumin was in males (1.02 ± 0.080 gm/dl) and females (2.11 ± 0.097 gm/dl) (Figs. 1 and 2). However, aspartate transaminase (AST) was not significantly different (*p *> 0.05) in male (966.50 ± 43.419 µl) and female (841.13 ± 123.890 µl).

The correlation matrix between morphometry and hematology is presented in Table 3. White blood cells (WBCs) count was significantly correlated with primary wing, body weight, tail length, body circumference, tarsal, metatarsal, and beak lengths. Red blood cells (RBCs) count was also significantly correlated with primary wing, body weight, tail length, and body circumference, while beak length was not significantly correlated with total length, wingspan, metatarsal, and head length. Hemoglobin (HGB) was significantly correlated with total length, body circumference, body length, primary wing, and body weight. There was no significant correlation between tail length, wing span, tarsal, metatarsal, and head length. Hematocrit (HCT) showed a significant correlation with body weight, primary wing, tarsal tail length, body circumference, beak length, total length, wingspan, metatarsal, and head length. MCV was significantly correlated with body weight, primary wing, tail length, body circumference, beak length, and significantly correlated with total length, tarsal, metatarsal, head length, and wingspan. MCHC was significantly correlated with body weight, primary wing, tarsal, tail length, and body circumference and not with head length, metatarsal, wing span, and total length. The rest of the parameters are presented in Table 3. RDW-SD and RDW-CV showed no significant correlation with all the morphometric parameters.

The correlation matrix between morphometry and plasma chemistry is presented in Table 4. Body weight was significantly correlated with urea, creatinine, protein, and albumin while not significantly correlated with ALT and AST. The total length and wingspan were not significantly correlated with all the biochemical parameters. The primary wing was significantly correlated with urea, creatinine, protein, and albumin while non-significantly correlated with ALT and AST. The body circumference was significantly correlated with urea, creatinine, protein, and albumin but was not significantly correlated with ALT and AST.

**Table 1.** Comparison of morphometric variables in male and female of common coot from Punjab (Pakistan).

Variable	Male (*n = 14*)		Female (*n = 18*)		Overall (*n = 32*)		
	Mean ± SEM	Range	Mean ± SEM	Range	Mean ± SEM	Range	*t (paired)*
Body weight (gm)	633 ± 10.7	567–680	444.56 ± 10.8	3,780–512	527 ± 18.4	378–680	12.17**
Total length (cm)	40.58 ± 0.6	38.00–44.60	39.39 ± 0.5	36.00–42.50	39.91 ± 0.4	36.00–44.60	1.57^NS^
Wing span (cm)	30.25 ± 0.2	29.20–31.50	29.15 ± 0.3	25.50–30.80	29.63 ± 0.2	25.50–31.50	2.67*
Primary wing (cm)	20.79 ± 0.2	20.00–21.90	19.07 ± 0.1	18.60–19.40	19.82 ± 0.2	18.60–21.90	9.20**
Tarsal length (cm)	6.46 ± 0.1	6.00–7.20	5.78 ± 0.1	5.20–6.20	6.08 ± 0.1	5.20–7.20	4.48**
Meta-tarsal length (cm)	8.92 ± 0.2	8.00–10	7.92 ± 0.1	6.50–8.60	8.36 ± 0.2	6.50–10	3.92**
Tail length (cm)	4.85 ± 0.1	4.50–5.40	7.46 ± 0.1	6.00–8.60	6.32 ± 0.2	4.50–8.60	–15.59**
Body circumference (cm)	27.81 ± 0.2	26.60–28.70	24.56 ± 0.03	22.00–26	25.98 ± 0.3	22.00–28.70	8.67**
Beak length (cm)	3.44 ± 0.1	2.80–4	3.75 ± 0.1	3.20-4	3.61 ± 0.1	2.80–4	–3.03**
Head length (cm)	3.36 ± 0.1	2.80–4	2.96 ± 0.1	2.40-4	3.13 ± 0.1	2.40–4	2.27*

*Significant (*p < *0.05).

**Highly significant (*p < *0.01).

Upper values indicated Pearson’s correlation coefficient; lower values indicated a level of significance at 5% probability. NS stand for non-significant.

**Table 2.** Comparison of hematological variables in male and female adult common coot sampled from Punjab (Pakistan).

Variable	Male (*n =* 10)			Female (n = 08)		Overall (*n* = 18)	
	Mean ± SEM	Range	Mean ± SEM	Range	Mean ± SEM	Range	*t* (Value)
WBC (10³/µl)	165.90 ± 7.15	129–190	244.50 ± 3.8	232–267	200.83 ± 10.4	129–267	–9.01**
RBC (106/µl)	3.03 ± 0.2	2.08–3.80	1.97 ± 0.1	1.70–2.13	2.56 ± 0.2	1.70–3.80	5.23**
HGB (gm/dl)	15.37 ± 0.4	13.40–16.90	13.45 ± 0.5	11.98–16.40	14.52 ± 0.4	11.98–16.90	2.90*
HCT (%)	48.32 ± 0.5	46.20–52.40	32.60 ± 0.5	31.09–35.40	41.33 ± 1.9	31.09–52.40	21.07**
MCV (fl)	180.80 ± 3.5	168.40–198.90	154.38 ± 4.1	135.60–17	169.06 ± 4.1	135.60–198.90	4.91**
MCH (pg)	64.25 ± 1.5	58.50–72.60	68.93 ± 2.8	56.50–78.50	66.33 ± 1.5	56.50–78.50	–1.60^NS^
MCHC (gm/dl)	76.72 ± 1.8	67.90–86.50	43.51 ± 1.2	38.50–48.50	61.96 ± 4.1	38.50–86.50	14.83**
PLT (×10³/µl)	12.80 ± 0.5	11–16	10.63 ± 0.4	9–12	11.83 ± 0.4	9–16	3.38**
RDW-SD (fl)	36.10 ± 0.6	33–38	38.13 ± 0.8	34–41	37 ± 0.5	33–41	–2.02^NS^
RDW-CV (fl)	5.41 ± 0.1	4.80–5.90	5.11 ± 0.1	4.60–5.40	5.28 ± 0.1	4.60–5.90	1.66^NS^
PDW (fl)	14.44 ± 0.2	13.60–15.20	13.09 ± 0.1	12.50–13.60	13.84 ± 0.2	12.50–15.20	5.53**
MPV (fl)	5.68 ± 0.1	5.20–6.20	5.06 ± 0.1	4.60–5.50	5.410.102	4.60–6.20	4.26**
PCT (%)	0.0 ± 0.0	0.0–0.0	0.02 ± 0.01	0.01–0.03	0.01 ± 0.002	0.00–0.03	–6.70**

Significant (*p < *0.05).

**Highly significant (*p < *0.01).

Upper values indicated Pearson’s correlation coefficient; lower values indicated a level of significance at 5% probability. NS stand for non-significant.

## Discussion

This study evaluated morphometry, hematology, and blood chemistry in common coots wintering at six main wetlands of the Punjab (Pakistan) during 2022–23. Morphometric studies on the common coot are few, though baseline information is required for interspecies and intraspecies variation and the effect of environmental conditions on the species [23]. In the current study, the morphometric measurements for common coots are very similar to those values reported by Grimmett et al. [24], Kiss [25], and Minias [23] in their studies. Minias [23] reported head length (74.31 ± 0.30 mm in male; 70.14 ± 0.27 mm in female), tarsal length (62.76 ± 0.48 mm in male; 58.14 ± 0.41 mm in female), and wing length (218.92 ± 0.87 mm in male; 206.60 ± 0.82 mm in female) in common coots. The values of tarsus length and primary wing length were similar or very close to the present study. The average body weight of the common coot was smaller, and the total length was larger in the present study as compared to the values reported by Minias [23] and Nouri et al [7]. These intra-species differences among the values of different morphometric parameters in the present study as compared to the literature may be attributed to the sensitivity of these traits to environmental changes such as temperature and nutrition [26,27].

**Table 3.** Pearson correlation coefficient matrix between haematology and morphometry in common coot.

Parameters	Body weight	Total length	Wing span	Pr. wing	Tarsal	Meta tarsal	Tail length	Body circumference	Beak length	Head length
WBC	–0.92**	–0.33	–0.39	–0.72**	–0.51*	–0.53*	0.86**	–0.85**	0.49*	–0.32
	0.0	0.19	0.11	0.001	0.03	0.02	0.0	0.0	0.04	0.2
RBC	0.67**	–0.11	0.21	0.69**	0.53*	0.33	–0.74**	0.77**	–0.53*	0.17
	0.003	0.66	0.41	0.002	0.02	0.20	0.0	0.0	0.02	0.50
HGB	0.58*	–0.32	–0.01	0.52*	0.3	–0.02	–0.67**	0.61**	–0.57*	0.31
	0.01	0.2	0.96	0.03	0.24	0.94	0.002	0.01	0.01	0.22
HCT	0.9**	0.13	0.34	0.83**	0.61**	0.43	–0.95**	0.91**	–0.64**	0.33
	0.0	0.62	0.18	0.0	0.007	0.07	0.0	0.0	0.004	0.18
MCV	0.68**	0.10	0.33	0.63**	0.42	0.15	–0.84**	0.75**	–0.51*	0.39
	0.002	0.69	0.18	0.005	0.08	0.56	0.0	0.0	0.03	0.11
MCH	–0.41	–0.17	0.01	–0.25	–0.17	–0.42	0.2	–0.26	–0.03	0.07
	0.09	0.49	0.97	0.31	0.49	0.08	0.43	0.31	0.91	0.79
MCHC	0.84**	0.07	0.32	0.85**	0.67**	0.41	–0.92**	0.87**	–0.64**	0.3
	0.0	0.78	0.19	0.0	0.002	0.09	0.0	0.0	0.01	0.23
PLT	0.49*	–0.26	–0.11	0.47*	0.34	0.1	–0.62**	0.54*	–0.26	0.17
	0.04	0.29	0.65	0.05	0.16	0.7	0.01	0.02	0.29	0.50
RDW-SD	–0.41	–0.14	0.09	–0.37	–0.29	–0.45	0.36	–0.33	0.16	0.20
	0.09	0.58	0.73	0.13	0.25	0.06	0.14	0.19	0.54	0.42
RDW-CV	0.31	–0.09	0.14	0.43	0.39	–0.04	–0.4	0.32	-0.29	0.35
	0.22	0.74	0.59	0.07	0.11	0.88	0.13	0.19	0.24	0.16
PDW	0.73**	0.05	0.25	0.75**	0.60**	0.3	–0.78**	0.69**	–0.49*	0.36
	0.001	0.85	0.32	0.00	0.008	0.23	0.00	0.002	0.04	0.14
MPV	0.64**	–0.11	0.21	0.67**	0.53*	0.18	–0.72**	0.62**	–0.44	0.248
	0.004	0.68	0.41	0.002	0.02	0.48	0.001	0.007	0.07	0.32
PCT	–0.81**	–0.38	–0.24	–0.73**	–0.6**	–0.51*	0.8**	–0.86**	0.52*	–0.22
	0.0	0.12	0.33	0.001	0.01	0.03	0.0	0.0	0.027	0.39

Upper values indicated Pearson’s correlation coefficient; lower values indicated a level of significance at 5% probability.

*Significant (*p < *0.05).

**Highly significant (*p < *0.01).

**Table 4.** Correlation matrix between morphometry and plasma chemistry.

Parameters	Urea	Create	ALT	AST	Protein	Albumin
Body weight	0.86**	0.82**	–0.44	0.39	–0.78**	–0.91**
	0.0	0.0	0.07	0.11	0.0	0.0
Total length	0.11	0.28	0.07	0.17	–0.27	–0.13
	0.66	0.3	0.8	0.50	0.29	0.6
Wing span	0.18	0.22	–0.28	–0.39	–0.32	–0.24
	0.49	0.39	0.27	0.11	0.2	0.34
Primary wing	0.75**	0.76**	–0.52*	0.19	–0.7**	–0.78**
	0.0	0.0	0.03	0.45	0.001	0.0
Tarsal	0.54*	0.58*	–0.43	0.05	–0.53*	–0.54*
	0.02	0.01	0.07	0.85	0.03	0.02
Metatarsal	0.43	0.52*	–0.26	0.09	–0.55*	–0.45
	0.08	0.03	0.29	0.73	0.02	0.06
Tail length	–0.90**	–0.84**	0.47*	–0.33	0.77**	0.91**
	0.0	0.0	0.05	0.18	0.0	0.0
Body circum.	0.81**	0.73**	–0.42	0.35	–0.83**	–0.9**
	0.0	0.001	0.08	0.15	0.0	0.0
Beak length	–0.54*	–0.46	0.35	–0.11	0.52*	0.6**
	0.02	0.06	0.16	0.66	0.03	0.01
Head length	0.13	0.32	–0.13	–0.2	–0.07	–0.25
	0.61	0.19	0.62	0.42	0.8	0.31

Upper values indicated Pearson’s correlation coefficient; Lower values indicated a level of significance at 5% probability.

*Significant (*p < *0.05).

**Highly significant (*p < *0.01).

In the current study, we found that male common coots are larger than female common coots, and these results are in line with the results of other studies [28,29]. Other morphometric parameters, including total length, wing length, tarsus length, and head length, are not reported yet; however, these parameters are reported in other species of coots. Male American coots (*Fulica americana*) showed larger values as compared to females [30,31]. The same trend was observed in the present study. Significant differences were also observed in the body weight of male and female crested coots (*Fulica cristata*) and common coots (*F. atra*) by Rubio, Ildefonso [31]. They reported males with larger body weight than females. The presently recorded values on 13 different morphometric variables on the common coot appear in the first record and can be used as baseline data on the species for future reference.

Blood is a specialized connective tissue that plays a vital role in physiological processes [32]. It is composed of special elements, including erythrocytes (RBCs), leukocytes (WBCs), and platelets, suspended within a fluid matrix and with plasma as the fluid portion [33–35]. Plasma biochemistry and hematology are useful tools for monitoring the bird’s health. They provide valuable information for ecological research. These tools provide a more integrative picture of the state of the animal than body mass indices alone [31,36]. The hematological values are used for diagnosis and monitoring of disease. Furthermore, these values are used for the evaluation of disease therapy or disease prognosis [37]. In addition, these values serve as reference values for different bird species. Various physiological factors can influence the hematology of healthy birds [38,39].

The hematological analysis indicates the state of health, metabolic profile, and health disorders [16,40]. The health status of an animal is indicated by hematological values, as many diseases and abnormalities are linked with the change in the hematology of an animal [41]. The analysis of hematology includes leukocytes or WBC counts that indicate the infection, HGB and HCT that evaluates the state of anemia, and the ratio of heterophil lymphocytes that indicate the stress state. These biological and physiological parameters, like weight and morphometry, are the indicators of health state [31,42].

The present study recorded all possible hematological variables in the common coot. Rubio, Ildefonso [29] reported HCT values (41.04% ± 5.8%), lymphocytes (67.85% ± 11.54%), heterophils (26.7% ± 10.41%), eosinophils (95% ± 2.62%), basophils (0.55% ± 1.23%), and monocytes (2.2% ± 3.56%) collected on 21 specimens of common coot (*F. atra*), which were close to the values suggested in the present study. Olayemi and Arowolo [43] reported values of different hematological variables in the Nigerian Duck. Hb concentration in Nigerian ducks was close to that reported for common coots, while MCH and MCV were lower in common as compared to Nigerian ducks, and MCHC was higher in common coots as compared to Nigerian ducks. The mean value of urea (mg/dl) in common coots was 21.72 ± 1.550 mg/dl, which was lower than that reported previously for common coots (43.3 ± 17.06) and higher than that in crusted coots *(F. cristata). *8.74 ± 3.35 [31].

## Conclusion

The morphometric analysis revealed significant differences between male and female coots in various parameters, highlighting sexual dimorphism, with males generally larger except for total length. Hematological examinations of blood samples unveiled distinct variations between the sexes in RBC count, HCT, MCV, MCH, MCHC, WBC count, and PLT, emphasizing the need for gender-specific considerations in such analysis. Notably, the correlation matrix established relationships between morphometric characteristics and hematological parameters, as well as between morphometry and plasma chemistry, shedding light on the interconnected nature of these physiological aspects. Furthermore, the blood chemistry analysis provided essential data on concentrations of urea, creatinine, albumin, total protein, AST, and ALT, offering a comprehensive overview of the metabolic and enzymatic status of the common coots. These findings contribute to our understanding of the health and physiological dynamics of this species in the studied region. In essence, this research not only advances the knowledge base on common coots but also underscores the importance of considering both morphometric and physiological aspects in ecological studies. The detailed insights provided by this study serve as a foundation for future investigations and contribute to the broader field of avian ecology.

## List of abbreviations

ALT, alanine transaminase; AST, aspartate transaminase; cm, centimeter; df, degree of freedom; DLC, differential leukocyte count; EDTA, Ethylenediaminetetraacetic acid; fl, femtoliter; gm/dl, grams per deciliter; Hb, hemoglobin; HCT, hematocrit; HGB, hemoglobin; MCH, mean corpuscular hemoglobin; MCHC, mean corpuscular hemoglobin concentration; MCV, mean corpuscular volume; MPV, mean platelet volume; µl, microliter; mg/dl, milligram per deciliter; *n*, total number; *p*, probability; PDW, platelet distribution width; PCT, procalcitonin; PCV, packed cell volume; PLTs, platelets; PVC, packed volume of cell; RBCs, red blood cells; RDW-CV, red cell distribution width–coefficient of variation; RDW-SD, red cell distribution width–SD; TEC, total erythrocytes count; TLC, total leukocyte count; WBCs, white blood cells.

## References

[1] Abro ZU, Kori SM, Qureshi AL, Mahessar AA (2018). Enhanced storage capacity and quality of Haleji and Hadero lakes connecting with Indus River for their sustainable revival. Pak J Sci Ind Res Ser A: Phy Sci.

[2] Shaffique S, Kang S-M, Ashraf MA, Umar A, Khan MS, Wajid M (2024). Research progress on migratory water birds: indicators of heavy metal pollution in inland wetland resources of Punjab, Pakistan. Water.

[3] Ullah I, Xue-Ying S, Qing-Ming W, Wen-You D, Khan TU, Rajpar MN (2024). Evaluation of water birds population trends, threats, and conservation status in selected wetlands of Pakistan. Pakistan J Zool.

[4] Kazam A, Sidra S, Ali Z, Ahmad R, Bilal A, Batool A (2024). Field validation of avian diversity at uchalli wetland complex: a ramsar site in Khushab, Pakistan. Pak J Zool.

[5] Yakovleva GA, Lebedeva DI, Bugmyrin SV (2021). Helminths of the Eurasian coot *Fulica atra* at Lake Ladoga coast (northwestern Russia). Wetlands.

[6] Chakraborty SK (2021). Diversity and conservation of wildlife associated with rivers: an eco-ethological analysis. Riverine ecology volume 2: biodiversity conservation, conflicts and resolution.

[7] Nouri N, Rachedi M, Lazli A, Samar F (2023). Study of the isotopic ecological niche of the common coot *Fulica atra* (lac tonga, north-eastern algeria). Appl Ecol Environ Res.

[8] Fu C, Kathait A, Lu G, Li X, Li F, Xing X (2021). A small vocal repertoire during the breeding season expresses complex behavioral motivations and individual signature in the common coot. BMC Zool.

[9] Zaman A, Rafique A, Jabeen F, Sultana T (2023). Diversity, abundance and seasonal assessment of wild birds in urban habitat of district Chiniot, Pakistan. Pak J Zool.

[10] Arshad M, Mehmood N, Muqadas H, Chaudhry J, Mustafa I, Khan MR (2014). Avifauna studies in co-relation with alteration in climatic patterns and hydrology of Uchalli Lake, Punjab, Pakistan. Pak J Zool.

[11] Rais M, Anwar M, Mehmood T, Hussain I (2011). Bird diversity and conservation at Kallar Kahar Lake with special reference to water birds. Pak J Zool.

[12] García J, Arizaga J, Rodríguez JI, Alonso D, Suárez‐Seoane S (2021). Morphological differentiation in a migratory bird across geographic gradients in mountains of southern Europe. J Biogeogr.

[13] Kadurumba OE, Ahmadu Y, Kadurumba C, Okafor OL, Okoli IC (2023). Characterisation and approaches to the conservation of the Nigerian local duck population: a Review. Agric Trop Subtrop.

[14] Verhoeven MA, Loonstra AJ, McBride AD, Tinbergen JM, Kentie R, Hooijmeijer JC (2020). Variation in egg size of black-tailed godwits. Ardea.

[15] Ritchison G (2023). Avian reproduction: timing, anatomy, and eggs. In a class of their own: a detailed examination of avian forms and functions.

[16] Kausar R, Anwar Z, Bashir R, Rehan S, Murtaza G, Usman M (2025). Seasonal variations in hematology of birds, in ecology of avian zoonotic diseases-new challenges.

[17] Shehzad A, Anjum K, Yaqub A, Yousaf MZ, Ditta SA, Naseer J (2022). Hematological status of avian species along a metal pollution gradient at Sialkot, Pakistan. Turk J Zool.

[18] Lawal JR, Ibrahim U, Biu A, Musa H (2024). Effects of avian malaria parasites infections on hematological and biochemical parameters in village chickens in Gombe state, Nigeria. J Vet Res Adv.

[19] Aslam M, Wajid M, Waheed A, Ahmad S, Jafar K, Akmal H (2021). Revision of some mensural measurements, food preference, and haematological parameters in breeding pairs of blue rock pigeon, Columba livia sampled from Punjab Pakistan. Braz J Biol.

[20] Rodriguez MD, Doherty PF, Piaggio AJ, Huyvaert KP (2021). Sex and nest type influence avian blood parasite prevalence in a high-elevation bird community. Parasit Vectors.

[21] Sripad K, Kowalli SKS, Metri RMR (2014). Serum biochemical and hematological profile of male, female and different age groups of Krishnavalley breed of cattle in Karnataka.

[22] Zia AB, Ali MA, Zeb MO, Shafiq U, Fida SR, Ahmed N (2016). Development of microfluidic lab-on-disc based portable blood testing point-of-care diagnostic device. In 2016 IEEE EMBS Conference on Biomedical Engineering and Sciences (IECBES), Kuala Lumpur, Malaysia.

[23] Minias P (2015). Sex determination of adult Eurasian Coots (*Fulica atra*) by morphometric measurements. Waterbirds.

[24] Grimmett R, Roberts TJ, Inskipp T, Byers C (2008). Birds of Pakistan.

[25] Kiss VA (2021). The morphometric analysis of Eurasian coot eggs (*Fulica atra*) under the local conditions from Câmpenești, North-Western Romania. Stud Univ Babes-Bolyai Biol.

[26] Yakubu A, Kaankuka F, Ugbo S (2011). Morphometric traits of Muscovy ducks from two agro-ecological zones of Nigeria. Tropicultura.

[27] Subasinghe K (2022). Avian morphometrics and climate change 2022.

[28] Riggs RA, Taylor AD, Thomson RC, Cowie RH (2019). Sexual dimorphism and seasonal variability of shield size in the endangered Hawaiian coot (*Fulica alai*). Waterbirds.

[29] Van Rees CB, Muñoz MA, Cooke SC, Reed JM (2021). Morphological differences in the island-endemic Hawaiian subspecies of the common gallinule *Gallinula galeata*. Pac Sci.

[30] López-Islas ME, Ibarra-Meza I, Ortiz-Ordóñez E, Favari L, Elías Sedeño-Díaz J, López-López E (2017). Biological responses of the American coot (*Fulica americana*), in wetlands with contrasting environmental conditions (Basin of México). J Toxicol Environ Health A.

[31] Rubio M, Ildefonso N, Agüera E, Almaraz P, De Miguel R, Escribano B (2014). Plasma biochemistry and haematology of crested coots (*Fulica cristata*) and common coots (*Fulica atra*) from Spain. Comp Clin Pathol.

[32] Singh AP, Maurya NK, Saxena R, Saxena S (2024). An overview of red blood cell properties and functions. J Int Res Med Pharm Sci.

[33] Garg G, Singh S, Singh AK, Rizvi SI, Sholl J, Rattan SI (2020). Characteristics of healthy blood. Explaining health across the sciences. Healthy ageing and longevity.

[34] Křížková V (2021). Blood and blood components, hematopoiesis, selected methods used in cytology, histology and hematology.

[35] Omonona A, Olukole S, Fushe F (2011). Haematology and serum biochemical parameters in free-ranging African side neck turtle (*Pelusios sinuatus*) in Ibadan, Nigeria. Acta Herpetol.

[36] Ferrer M, Evans R, Hedley J, Hollamby S, Meredith A, Morandini V (2023). Plasma chemistry and hematology reference values in wild nestlings of White-tailed Sea Eagles (*Haliaeetus albicilla*): effects of age, sex and hatching date. J Ornithol.

[37] Youssef IM, Khalil HA, Jaber FA, Alhazzaa RA, Alkholy SO, Almehmadi AM (2023). Influence of dietary mannan-oligosaccharides supplementation on hematological characteristics, blood biochemical parameters, immune response and histological state of laying hens. Poult Sci.

[38] Zapletal D, Kudělková L, Šimek V, Jakešová P, Macháček M, Straková E (2017). Haematological indicators in hybrid mallard ducks (*Anas platyrhynchos*) with regard to the use of meal from whole white lupin seeds in their diet. Acta Vet Brno.

[39] Kral I, Suchý P (2000). Haematological studies in adolescent breeding cocks. Acta Vet Brno.

[40] Kuzmina N, Petrov OY, Semenov V, Boronin V (2021). The effect of an antioxidant on the hematological profile of birds. IOP Conf Ser: Earth Environ Sci.

[41] Orakpoghenor O, Markus TP, Ogbuagu NE, Enam SJ, Oladele SB, Abdu PA (2021). Age-dependent variations in haematological and serum biochemical parameters of domestic pigeons (*Columba livia domestica*). Heliyon.

[42] Lobato DN, Braga ÉM, Belo NDO, Antonini Y (2011). Hematological and parasitological health conditions of the Pale-breasted Thrush (*Turdus leucomelas*) (Passeriformes: Turdidae) in southeastern Brazil. Zoologia.

[43] Olayemi F, Arowolo R (2009). Seasonal variations in the haematological values of the Nigerian Duck (*Anas platyrhynchos*). Int J Poul Sci.

